# Training in critical care cardiology: making the case for a standardized core curriculum

**DOI:** 10.1186/s13054-023-04649-6

**Published:** 2023-10-02

**Authors:** Leonhard Binzenhöfer, Holger Thiele, Enzo Lüsebrink

**Affiliations:** 1 Department of Medicine I, LMU University Hospital, LMU Munich, Marchioninistraße 15, 81377 Munich, Germany; 2 DZHK (German Center for Cardiovascular Research), Partner Site Munich Heart Alliance, Munich, Germany; 3 Department of Internal Medicine/Cardiology and Leipzig Heart Science, Heart Center Leipzig at University of Leipzig, Leipzig, Germany

## Introduction

The implementation of interventional cardiac procedures and mechanical circulatory support (MCS) devices has led to a fundamental transformation of critical care cardiology. Today, both technological advances and a more nuanced understanding of outcomes allow for the delivery of various complex procedures in high-risk settings and to older patients with severe comorbidities [1]. Considering the expanding wealth of knowledge and skill required for the management of such complex cases, cardiac intensive care unit (CICU) teams face unprecedented challenges that need to be addressed within the educational pathway of critical care cardiology. In this article, we outline key training elements for aspiring cardiac intensivists and advocate for a contemporary core curriculum that allows for the integration of these elements into an overarching sub-specialization concept in critical care cardiology.

## Cardiovascular fellowship: challenging first steps in critical care training

Modern cardiovascular fellowship programs typically include a 6-month general or CICU rotation, which may be prolonged depending on individual preferences. The goal for this period set forth by the Core Cardiovascular Training Statement (COCATS) 4 Task Force 13 and the 2020 European Society of Cardiology (ESC) curriculum for the cardiologist is to manage the majority of cardiovascular patients in intensive care settings [2, 3].

Many prerequisites for CICU patient care are taught as part of rotations in cardiac imaging, electrophysiology, and emergency medicine. Core training components during the first CICU rotation comprise advanced hemodynamic monitoring, airway and respiratory management, basic circulatory shock management, cardiac arrest algorithms and post-resuscitation care, sedation, monitoring of neurological function, infection control, and management of multi-organ dysfunction. Extracurricular seminars, such as the in-depth courses on acute myocardial ischemia, hypertension, and organ transplantation offered by the European Society of Intensive Care Medicine (ESICM) or the Society of Critical Care Medicine (SCCM) can solidify theoretical knowledge and foster interaction with peers. In general, early scholarly activity and participation in (inter)national meetings is encouraged by teaching institutions and the respective societies.

In recent years, we have been witnessing a growing interdependence of interventional and critical care cardiology. Coronary angiography has become a cornerstone of myocardial infarction-related cardiogenic shock management. Beyond that, patients presenting with acute decompensated valvular dysfunction, massive pulmonary embolism, arrhythmia-related hemodynamic instability, or intracardiac shunts may qualify for emergent transcatheter procedures. While expert multidisciplinary teams formulate the most promising treatment approach, CICU fellows play a central role for its successful implementation. Following interventional procedures, the access site vasculature, potential thromboembolic complications, arrhythmias, and end organ function require constant vigilance. Echocardiographic visualization of implanted devices and assessment of their function together with general hemodynamic changes needs to be performed reliably and specific procedure-related complications must be recognized and addressed in a timely manner.

Apart from these major advances in interventional cardiology, contemporary cardiogenic shock management became a complex endeavor, particularly due to the widespread usage of MCS devices for patients with severe hemodynamic compromise. Although based on recent data the future role of veno-arterial extracorporeal membrane oxygenation (VA-ECMO), Impella, and other MCS options is uncertain [4, 5], cardiovascular fellows will nonetheless be confronted with the responsibility of handling these devices safely, detecting complications early, and initiating effective countermeasures.

## Sub-specialization in critical care cardiology

After specialization in general cardiovascular or intensive care medicine, accreditation in critical care cardiology necessitates exposure to CICU cases for an extended period of time, ideally within a dedicated sub-specialization program at an accredited tertiary institution. There is consensus on the relevance for offering structured up-to-date training pathways to prepare for the staggering challenges in cardiovascular intensive care and allow for standardized quality assessment. However, sub-specialization models and accreditation standards still vary substantially with respect to training time and curricula.

Managing CICU cases independently requires a broad practical skillset, general intensive care expertise, and in-depth knowledge regarding cardiac illnesses and the respective treatment options. Clinical training elements in this career stage include decision-making regarding interventional procedures in high-risk settings, intimate knowledge and skillset required for comprehensive peri-interventional care, familiarity with all forms of circulatory shock and advanced MCS device management including cannulation/decannulation, weaning, LV venting modalities, as well as combined MCS. A contemporary CICU core curriculum should aim to distinguish the milestones for pursuing a career in critical care cardiology from the scope of critical care training that applies to a broader silo of cardiovascular fellows.

Beyond patient care, critical care cardiologists must be proficient in many other rapidly evolving areas of competence, such as IT-systems, billing, professional communication skills particularly in end-of-life scenarios, and the legal framework of clinical trials. Palliative care, the allocation of limited financial resources and clinical trial participation involve demanding ethical considerations. Contemporary CICU training programs should incorporate these additional skills as part of a well-rounded education in intensive care medicine.

Teaching the vast theoretical and practical armamentarium of critical care cardiology in context of recent advances in the field calls for an updated core curriculum covering all aspects of critical care training for cardiovascular fellows as well as aspiring CICU specialists. Apart from integrating the ever-expanding list of competences, a holistic training concept must be flexible enough to allow for personalization of the candidate’s career pathway. To incentivize fellows to pursue the lengthy and demanding training required to become an expert in critical care cardiology, a modular curriculum seems to be the most applicable basis for tailoring their education to prior professional experience and personal wishes, including the desire for maternity/paternity leave, and international workplace mobility. Importantly, training programs should be conceptualized to allow for interruption of clinical training for research purposes and hybridization with sub-specialization programs in interventional cardiology or advanced heart failure [6].

## Conclusion

The evolution of modern critical care cardiology has generated a burgeoning demand for specialists in this domain of cardiovascular medicine, and by extension, a need for an updated concept for sub-specialization that is both comprehensive and flexible. Despite the general consensus that there is a need for such training standards, dedicated sub-specialization tracks or advanced fellowships in cardiac intensive care are rarely available and heterogeneous, which continues to stunt the progression of leaders in the field [7]. A contemporary core curriculum endorsed by the major intensive care societies may incentivize fellows to pursue a career in critical care cardiology, encourage teaching institutions to offer a tangible educational perspective, and ultimately improve the quality of care for critically ill patients.

## Data Availability

Not applicable.
